# Overcoming the HIV Pre-Exposure Prophylaxis Uptake Barrier With A Health Action Process Approach and Conditional Economic Incentives Among Men Who Have Sex With Men in 3 Chinese Cities: Protocol for a Randomized Controlled Trial

**DOI:** 10.2196/82123

**Published:** 2026-04-20

**Authors:** Hao Lin, Mojun Ni, Chun Chang, Feng Sun, Jinghua Li, Omar Galárraga, Phoenix Kit-Han Mo, Wangnan Cao

**Affiliations:** 1 School of Public Health, Peking University Health Science Center, 38# Xueyuan Road, Haidian District, Beijing, 100191, China, 86 15210817312; 2 Department of Public Health and Medicinal Administration, Faculty of Health Sciences, University of Macau, Macau, China; 3 School of Public Health, Center for Global Public Health, Brown University, Providence, RI, United States; 4 School of Public Health and Primary Care, The Chinese University of Hong Kong, Hong Kong, China (Hong Kong)

**Keywords:** HIV, pre-exposure prophylaxis, men who have sex with men, health action process approach, economic incentive, randomized controlled trial, China

## Abstract

**Background:**

Despite high pre-exposure prophylaxis (PrEP) awareness and willingness among Chinese men who have sex with men (MSM), actual uptake remains critically low. The health action process approach (HAPA) is a 2-phase theory that addresses both the motivational and volitional phases of health behavior change, making it suitable for tackling the intention-behavior gap in PrEP use. In China, where PrEP is not covered by national health insurance, financial barriers further hinder uptake. Group-based conditional economic incentives (GCEIs) represent an innovative strategy to address cost-related disincentives by leveraging peer influence.

**Objective:**

This randomized controlled trial aims to evaluate the effectiveness of HAPA-driven interventions, both alone and combined with GCEI, to enhance PrEP initiation and adherence among high-risk, PrEP-naive MSM in China.

**Methods:**

High-risk HIV-negative MSM naïve to PrEP were recruited via community-based organizations in 3 Chinese cities. Participants underwent individual randomization to 3 arms: (1) HAPA-based cognitive behavioral intervention featuring tailored WeChat messages and 2 structured motivational interviews targeting stage-specific barriers; (2) combined HAPA intervention with GCEI linked to peer group initiation rates, incorporating facilitated offline group activities; or (3) standard care control receiving nonpersonalized HIV prevention information. The physician-prescribed PrEP initiation rate at 3 months serves as the primary outcome. Secondary outcomes include PrEP adherence, HIV risk perception, PrEP-related stigma, and intervention acceptability. Analysis follows intention-to-treat principles using mixed-effects models.

**Results:**

The study protocol received ethics approval in December 2024 and was funded in December 2025. As of August 2025, 3 community-based organizations in Mianyang, Zibo, and Hohhot have agreed to participate. Preliminary testing has led to refinements in the WeChat mini-program, questionnaires, and motivational interview scripts. Data collection commenced in October 2025 and is projected to be completed by August 2026. As of February 2026, baseline recruitment is complete, with 381 eligible participants enrolled and randomized. The baseline characteristics were balanced across the 3 arms. Outcome data will be collected after the intervention. Data analysis is ongoing, with full results expected to be published before December 2026.

**Conclusions:**

This study rigorously tests a novel intervention integrating the HAPA with a GCEI model. By addressing both individual cognitive-behavioral barriers and financial disincentives within a supportive group context, this approach offers a potentially scalable strategy to bridge the PrEP uptake gap in China. Findings will provide critical evidence on combining theory-driven behavioral techniques with peer-group economic incentives for HIV prevention.

## Introduction

The burden of AIDS remains substantial. It is estimated that 0.7% (95% uncertainty interval 0.6‐0.8%) of adults aged 15‐49 worldwide are living with HIV, with a higher prevalence of 7.5% among men who have sex with men (MSM) [1]. In China, by the end of 2023, there were 1.3 million people living with HIV or AIDS, with a cumulative death toll of 458,000 [2]. The incidence of new HIV infections among MSM in China is 5.6 per 100 person-years [3]. The risk of HIV transmission remains significant, necessitating effective measures among groups at high risk, such as MSM, to control the spread of the epidemic [4].

Pre-exposure prophylaxis (PrEP) is an efficient biological prevention method to prevent HIV infection [5]. In most high-income countries, the overall PrEP usage rate among high-risk groups ranges from 28% to 80% [6]. Currently, international recommendations for MSM include oral tenofovir disoproxil fumarate or emtricitabine and long-acting injectable-PrEP (LAI-PrEP), both of which have been proven to effectively reduce the risk of HIV infection [3,4]. China has made significant efforts to promote PrEP among high-risk groups, such as launching pilot projects and approving generic medicine [7]. However, low willingness to use oral PrEP among key populations [5], stemming from cognitive biases, leads to low PrEP adoption despite potentially high demand [8]. There are over 12 million MSM in China [9], with PrEP awareness rates ranging from 41% to 86% [10,11], and approximately 65.8% to 84.9% of MSM willing to accept PrEP [12,13]. A survey of 2388 MSM across 6 Chinese cities showed that only 13.9% had used PrEP in 2022 [14]. Factors such as low health literacy, low behavior control, distrust in PrEP efficacy, lack of knowledge about PrEP, high costs, discrimination, and stigma are major barriers to PrEP use despite high demand [15,16]. Among those who initiate PrEP, its protective efficacy depends on sufficiently high adherence [17,18]. PrEP adherence rates among MSM in China range from 29.9% to 41.1% [19,20]. For LAI-PrEP, the long-acting injectable cabotegravir was approved in China in May 2024, and its usage intention and behavior among key populations are still being explored [7].

Behavioral interventions to promote PrEP among groups at high risk need to focus on increasing initiation rates before initiation and improving adherence after initiation. Existing interventions to promote PrEP initiation among MSM include PrEP health education [21], motivational structured interviews [22], MSM peer education and guidance [23], and mobile health technology interventions [24]. In China, smartphone-based interventions and app-based PrEP initiation interventions have shown some effectiveness, with PrEP initiation rates ranging from 65% to 89% [25]. The health action process approach (HAPA) is a 2-phase theory that divides behavior into the preintentional motivational phase (focusing on promoting the generation of behavioral intention) and the postintentional volitional phase (focusing on translating intention into behavior, maintaining behavior, and managing relapse) [26]. The HAPA phase model can effectively address the high willingness–low usage gap in PrEP and is particularly suitable for explaining and measuring low willingness, low usage rates, low adherence, and low reinitiation rates after discontinuation at different PrEP stages. Chan et al [27] used motivational structured interviews to improve PrEP initiation rates, with the core of the intervention being to explore and strengthen individuals’ intrinsic motivation to use PrEP and improve self-efficacy through situational simulation. Clarifying the suitability of PrEP and risk behavior is key to further effective PrEP intervention [28].

In China, where PrEP medications are not yet covered by national health insurance, financial barriers significantly hinder uptake among MSM despite high willingness. The out-of-pocket cost for oral PrEP (eg, approximately US $5.5 per on-demand dose) poses a critical obstacle, particularly for low-income individuals. In China, the monthly cost of domestically produced generic oral PrEP is approximately ¥200‐300 (US $28‐$42); for on-demand dosing, the cost per use is about ¥40 (US $5.6). This context underscores the need for innovative strategies to mitigate cost-related disincentives. Group-based conditional economic incentives (GCEIs) offer a promising solution by leveraging behavioral economics principles. GCEI capitalizes on peer influence and shared accountability, addressing both financial and social barriers [29,30]. For MSM, whose health behaviors are often shaped by community norms, GCEI can amplify motivation through collective goal-setting (eg, group-level initiation targets) and tiered rewards (eg, US $22 for 80%‐100% group adherence). Prior studies demonstrate GCEI’s efficacy in improving antiretroviral therapy adherence among youth, suggesting translatability to PrEP promotion [31-33]. By integrating financial incentives with peer dynamics, GCEI aligns with China’s need for scalable, cost-sensitive interventions to bridge the intention-behavior gap in PrEP uptake.

Existing research on PrEP uptake among Chinese MSM has largely relied on cross-sectional analyses that identify barriers but fail to translate findings into effective interventions. These studies consistently highlight structural challenges—particularly financial constraints in China’s self-pay PrEP model and pervasive stigma surrounding both HIV prevention and same-sex behaviors—yet proposed solutions rarely account for the cultural and logistical realities of this population. Our study addresses these limitations through an intervention model that combines theory-driven behavioral strategies with pragmatic delivery methods tailored to the Chinese context. The use of WeChat-based eHealth platforms proves particularly strategic, as it capitalizes on near-universal smartphone penetration among Chinese MSM while circumventing stigma through discreet, asynchronous engagement. This approach recognizes that target users may avoid clinic-based services due to privacy concerns, yet actively participate in virtual communities where health information is exchanged. By embedding the intervention within a familiar digital ecosystem and incorporating group-based incentives that leverage existing social networks, the design intentionally works with—rather than against—the lived experiences of Chinese MSM. The result is an implementation framework that simultaneously addresses individual behavioral determinants and systemic barriers, offering a realistic path to scaling PrEP access in stigma-affected populations.

This study aims to evaluate the effectiveness of HAPA-based interventions, alone and combined with GCEI, in improving PrEP initiation and adherence among Chinese MSM at high risk for HIV infection. We hypothesize that both intervention arms (HAPA and HAPA+GCEI) will outperform the standard care control in promoting PrEP initiation, with the combined HAPA+GCEI arm expected to yield the greatest efficacy.

## Methods

### Study Design

This study uses a randomized controlled trial design. The MSM-friendly community-based organizations (CBOs) are selected, and the participants are randomly allocated to the following three arms: (1) HAPA-based cognitive intervention arm, (2) the HAPA+ conditional economic incentive combined intervention arm, and (3) control arm. The intervention will last for 3 months, during which baseline data and 2 follow-up assessments will be collected. The first follow-up will occur at the conclusion of the intervention, while the second follow-up will take place three months postintervention. We will assess PrEP uptake before and after the intervention and between different study arms. We used the SPIRIT 2025 checklist to ensure completeness of the trial protocol reporting (Checklist 1).

### Study Population and Participant Recruitment

The study population is sourced from 3 MSM-friendly CBOs located in different regions. The inclusion criteria for community organizations are: (1) the organization must meet the sample size requirements for the project; (2) there must be a willingness to collaborate, making PrEP promotion a work priority; and (3) the organization should have a good partnership with local hospitals or centers for disease control and prevention to coordinate with disease control specialists and HIV specialists. Community collaborators participate in project training, assist with participant recruitment, support baseline and follow-up surveys, and help with quality control. Participants included in this study must meet the following criteria: (1) aged 18 years or older; (2) assigned male sex at birth and have had sexual intercourse with men in the past 6 months; (3) HIV negative at the start of the study; (4) no prior PrEP using experience; (5) willing to participate in the PrEP intervention and voluntarily consent to complete data collection and follow-up according to the assigned protocol; (6) classified as high risk for HIV infection in the past 6 months as suggested by the “Expert Consensus on HIV Pre-Exposure Prophylaxis in China (2023)” [7]. Participants are excluded if they meet any of the following criteria: (1) HIV positive, or unaware of their HIV status and refuse to undergo HIV testing; (2) have a health condition that makes PrEP unsuitable; and (3) have cognitive impairments or other severe illnesses that may affect the informed consent process.

The sample size calculation was based on the formula for an individually randomized controlled trial, assuming a 2-sided significance level (*α*) of .05 and type II error rate (*β*) of .10 (90% power). Based on extant literature and a 2021 multicenter PrEP survey in China indicating a median current PrEP usage rate (*p₁*) of 5% among MSM, the target post-intervention usage rate (*p₂*) was set at 20%. This yielded a minimum requirement of 100 participants per group. Accounting for an anticipated 15% attrition rate, the sample size was increased to 126 participants per group, resulting in a total target enrollment of 378 participants (126 per group) across 3 CBOs.

The randomization sequence was generated by an independent statistician using simple randomization. The sequence was then integrated into the backend of the customized WeChat mini-program. After a participant completed the baseline survey and was confirmed eligible, the system automatically executed the group assignment. Allocation concealment was strictly implemented. After a participant completed the baseline assessment and was confirmed eligible, the system automatically executed the group assignment based on the preloaded, concealed sequence. Until this point of allocation, neither the participant, the research coordinator enrolling the participant, nor the outcome assessors could access or predict the upcoming assignment. The allocation is only revealed to the system and the intervention coordinator after the participant’s baseline data is irrevocably locked and enrollment is finalized. The participants are blinded to group assignment. Outcome assessors and data analysts remain blinded to group assignment throughout the trial. They have access only to deidentified participant data coded with unique study IDs, without any information linking the ID to the intervention arm.

### Intervention

#### Tailored Health Messages

Participants receive a 3-month mobile health intervention guided by the HAPA. Tailored messages are developed using behavior change techniques based on theoretical guidance [34]. Three times weekly, the personalized health message is randomly delivered to participants via the WeChat mini-program. These messages are drawn from an information library designed based on HAPA theory, expert input, and literature review, and are tailored to the participant’s current PrEP using status. For example: participants exhibiting low risk perception receive messages incorporating vivid illustrations of potential health risks associated with their PrEP adherence status, using real-life case studies and epidemiological data to enhance awareness. Participants with low self-efficacy receive messages incorporating techniques such as providing success stories of similar individuals overcoming adherence challenges and offering step-by-step guidance to bolster confidence in maintaining the PrEP regimen.

#### Brief PrEP Motivational Interviewing

The brief motivational interviewing sessions are conducted by experienced professionals with demonstrated expertise in both PrEP provision and motivational interviewing techniques. These 1-on-1 sessions (delivered face-to-face or online) are tailored to each participant’s specific stage within the PrEP motivational cascade, such as objective identification, PrEP precontemplation, PrEP contemplation, PrEP preparation, PrEP action and initiation, and PrEP maintenance. Content is individualized to address specific barriers currently present, informed primarily by findings from the participant’s most recent assessment phase. This targeted approach aims to resolve impediments to PrEP engagement through structured, theory-guided dialogue.

#### Conditional Economic Incentives

Participants in the combined intervention arm also receive additional peer GCEIs on top of the HAPA intervention. Incentive information is communicated immediately at the start of the intervention. The incentive amounts are determined at the end of the 3-month intervention by the researchers based on the overall completion status of each participant and their peer group according to predetermined conditions.

Participants are formed into peer groups of approximately 6 individuals. These groups participate in structured in-person sessions. Activities are designed to include: introductions and icebreakers, PrEP knowledge quizzes, partner dialogues, “Voicing My Barriers” roundtable discussions, and PrEP scenario role-playing. The objectives are to foster trust, enable deeper peer connection, enhance group belonging, strengthen HIV prevention awareness, identify and overcome barriers to PrEP use, and ultimately increase PrEP initiation rates. Six participants form a peer group, and the economic incentive amount is based on the average completion level of the group. Group-level economic incentives are stepwise reduced based on the group’s average PrEP initiation rate. Participants qualify for US $22 if their group achieves an 80%‐100% rate, US $11 for a 50%‐79% rate, and US $0 for rates below 50%. PrEP initiation is operationally defined as the documented provision of a physician-issued prescription.

The WeChat mini-program automatically records intervention process evaluation indicators, including: (1) whether each participant has read the information messages; (2) the reading time for each message; and (3) each participant’s judgment of whether the information was helpful.

### Control

Participants receive nonpersonalized HIV prevention information, including free HIV testing and condom use, following the guidelines provided by the Chinese Center for Disease Control and Prevention and the operational standards of the community organizations, with twice-weekly information push notifications.

### Outcomes

The primary outcome indicator of this study is the rate of PrEP initiation, based on formal PrEP prescriptions issued by doctors. The secondary outcome indicators include medication adherence rates after initiating PrEP (based on self-reported adherence and the medication calendar in the mini-program), intention to pay for PrEP (for 3 types of PrEP), PrEP stigma, changes in condom use, and changes in number of sexual partners before and after PrEP use.

### Measurements and Follow-Ups

For the questionnaire survey, participants are informed about the study’s objectives, content, and procedures, and they sign an electronic informed consent form. The following baseline assessment questionnaire is conducted via a mini-program:

1. Demographic characteristics: Includes age, ethnicity, education level, marital status, employment status, income level, and sexual orientation
2. Sexual behavior characteristics: Includes age at first sexual intercourse, types and number of sexual partners, sexual roles, condom use, HIV testing, and whether they have paid or have been paid for sex
3. PrEP usage intentions and behaviors: Includes a PrEP knowledge assessment questionnaire, awareness rates and sources of information about PrEP, willingness to use and pay for PrEP, past PrEP usage, types of PrEP, frequency of use, and willingness to use different types of PrEP
4. HAPA-based PrEP cognition scale: Includes self-efficacy for initiating, maintaining, and resuming PrEP use, positive outcome expectations for PrEP, negative outcome expectations for PrEP, perceived HIV risk, willingness to use PrEP, and action plans for initiating and maintaining PrEP use. This scale has been developed and validated among the MSM population, comprising 9 dimensions and 44 items, using a 5-point Likert scale from 1 (“strongly disagree”) to 5 (“strongly agree”), with a total Cronbach α of 0.882 [35]
5. PrEP Stigma Scale: Uses a 10-item HIV PrEP Stigma Scale, which has been validated in multiple countries, eg, “using PrEP will lead to negative evaluations by others.” The scale uses a 5-point Likert score from 1 (“strongly disagree”) to 5 (“strongly agree”), with higher scores indicating greater PrEP stigma, and a Cronbach α of 0.880 [36].

Follow-ups are conducted at the end of the intervention and 3 months later. The follow-up questionnaire builds upon the baseline assessment, focusing on the following key areas:

1. Intervention process and satisfaction evaluation: This evaluates participants’ experience and satisfaction with the mobile health intervention platform (the WeChat mini-program), including usage frequency, overall satisfaction, willingness to recommend it, and specific feedback on its ease of use, interface design, information resources, medication reminders, recording functions, and data privacy protection.
2. Risk compensation behavior assessment: It investigates changes in participants’ sexual behavior patterns during the intervention to assess the possibility of risk compensation, including changes in the frequency of same-sex intercourse, number of male sexual partners, and condom use frequency compared to baseline.
3. PrEP uptake status: Based on the current PrEP status at follow-up, it further explores specific reasons for noninitiation or discontinuation, sources of medication acquisition, methods of recording medication use, episodes of treatment interruption, occurrence of adverse drug reactions, partner awareness, psychological experiences related to PrEP use, and willingness to recommend it.
4. Dynamic measurement of PrEP-related cognition: It reapplies key items from the validated HAPA-based PrEP cognition scale to assess changes in postintervention cognition, including self-efficacy for PrEP initiation and maintenance, positive and negative outcome expectations, perceived HIV risk, and action planning.
5. Reassessment of PrEP decision and payment willingness: It re-evaluates participants’ perception of pricing and willingness to use different PrEP regimens at market prices, as well as their views on potential models for cost-sharing.
6. Follow-up on PrEP perceived stigma and MSM group cohesion: It uses a brief mental health scale to assess emotional states over the past week, remeasures PrEP perceived stigma levels, and evaluates changes in perceived cohesion within the MSM community.

The sexually transmitted infections testing data (HIV, syphilis, hepatitis B, and hepatitis C) from participants at baseline and during 2 follow-up visits are collected. All tests are performed using standardized diagnostic kits (Wondfo Biotech) according to the manufacturer’s protocols. Serological results are recorded as binary outcomes (positive or negative). For participants who initiated PrEP during the study, adverse drug reaction evaluations are integrated throughout the PrEP medical service process. A formal safety stopping rule is prespecified. The trial will be halted immediately if the independent Data and Safety Monitoring Board identifies clear, serious, and related adverse events attributable to the study procedures that pose unacceptable risks to participants, or if new, definitive external evidence emerges that renders the trial unethical or obsolete.

### Preliminary Testing and Modifications

Preliminary testing with key stakeholders enabled critical refinements to study instruments and procedures, including substantial enhancements to the WeChat mini-program’s navigation flow to facilitate access to PrEP information and intervention components, along with added privacy protection features such as local data storage and optional fingerprint authentication, while message delivery timing was optimized based on observed user activity patterns. Questionnaire modifications involved clarifying wording in the PrEP Stigma Scale to better differentiate between perceived and internalized stigma, and supplementing HAPA-based scales with concrete examples to improve comprehension of abstract constructs like self-efficacy.

The motivational interview protocol is finalized after cultural appropriateness testing, resulting in standardized prompts for exploring financial and structural barriers specific to Chinese MSM populations, as well as incorporating guidance for discussing newly approved LAI-PrEP options, collectively strengthening the intervention’s relevance and implementation fidelity for the target population.

### Ethical Considerations

The study protocol was approved by the Ethics Commission of Peking University Health Science Center (IRB00001052-24167) on December 22, 2024. Prior to study initiation, all participants are fully informed about the study objectives, procedures, potential risks, and their rights, and provide written informed consent. Participants receive a compensation of US $2.8 (approximately CNY 20) for completing each questionnaire at baseline and follow-up assessments. The study does not provide free PrEP medication; participants are required to purchase medications at market prices. To protect participant privacy and confidentiality, several measures have been implemented: data collection uses a customized WeChat mini-program featuring local data storage and optional fingerprint authentication for enhanced privacy; all questionnaires are administered anonymously and linked only via a unique study ID; and sexually transmitted infections test results (HIV, syphilis, hepatitis B, and hepatitis C) are recorded solely as binary (positive or negative) outcomes without any personally identifiable information.

### Statistical Analysis

#### Descriptive Analysis of Baseline Data and Intergroup Comparisons

For numeric variables such as PrEP usage intentions and HAPA component scores, the mean and SD (or median and IQR) will be reported based on the data distribution. Mann-Whitney tests or *t* tests will be used to compare the baseline data balance between the intervention and control groups according to the data distribution type. Categorical variables such as PrEP initiation and adherence will be described using rates, and chi-square tests or Mann-Whitney tests will be used to compare the baseline data balance between the intervention and control groups.

#### Evaluation of Intervention Effectiveness

This study will use mixed-effects models to evaluate the effectiveness of primary and secondary outcome indicators. Different evaluation models will be selected based on the type of outcome indicators. Individual participants will be the unit of analysis. For the evaluation of categorical variable effects, a generalized mixed linear model will be used, reporting the adjusted odds ratio. For continuous variable effects, a mixed linear model will be used, reporting the adjusted mean differences between groups. The effectiveness evaluation will adhere to the intention-to-treat principle, including all randomized participants in the analysis. Stratified analysis will be conducted based on participants’ age, education level, income level, sample source, and city characteristics to explore evidence of effects related to these various characteristics. Statistical analyses will be performed using R (R Foundation for Statistical Computing) and SPSS software (IBM Corp), with a *P* value<.05 considered statistically significant. The study assumes that missing values are randomly distributed, and missing data will be handled using maximum likelihood estimation. Sensitivity analyses will be conducted after imputing missing values using multiple imputation methods.

## Results

**Figure 1.**
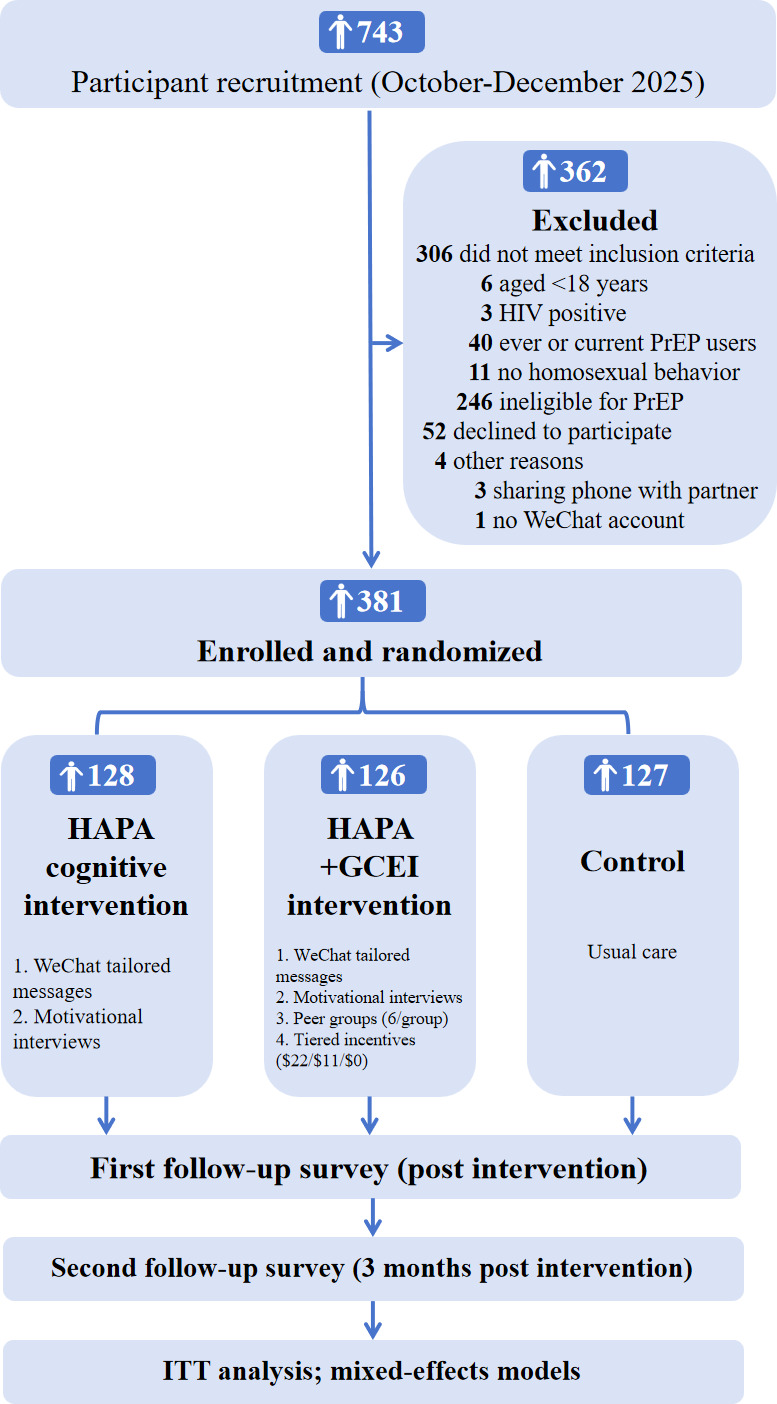
Participant flowchart (status as of February 2026: baseline assessment and randomization completed; interventions are ongoing). GCEI: group-based conditional economic incentive; HAPA: health action process approach; ITT: intention-to-treat.

The trial is a 3-arm, parallel-group, randomized controlled trial conducted in collaboration with 3 CBOs located in Mianyang (Sichuan Province), Zibo (Shandong Province), and Hohhot (Inner Mongolia), China. The study protocol was funded in December 2025. Key operational phases, from recruitment to analysis, with specific dates and participant flow, are presented in Figure 1.

### Participant Recruitment

Participant recruitment and screening were conducted from October to December 2025. During this period, 743 eligible MSM from the 3 CBOs were initially identified and assessed. Of these, 362 individuals were excluded for the following reasons: failure to meet predefined inclusion criteria (n=306), declining to participate (n=52), or other logistical reasons (n=4). Consequently, 381 participants provided written informed consent and were formally enrolled in the study. These 381 participants were randomly allocated into 1 of 3 study arms using a computer-generated sequence: HAPA-based cognitive behavioral intervention group (n=128), HAPA plus GCEI intervention group (n=126), and control group (n=127).

### Participant Baseline Characteristics

As shown in Table 1, the baseline sociodemographic characteristics of the participants were well-balanced across the 3 study groups. Specifically, the average ages across the 3 groups were similar, with the HAPA cognitive intervention group averaging 34.5 (SD 9.9) years, the HAPA combined conditional economic incentive intervention group averaging 35.4 (SD 10.6) years, and the control group averaging 34.3 (SD 10.6) years (*F*_2,378_=0.411; *P*=.66). The geographical distribution of participants among the 3 recruitment cities (Mianyang, Zibo, Hohhot) was also balanced and consistent across all groups (*χ*²_4_=0.205; *P*=.96).

**Table 1.** Baseline sociodemographic characteristics of participants (N=381).

Characteristic	Group			*F*/chi-square (*df*)^a^	*P* value
	HAPA^b^ cognitive intervention (n=128)	HAPA+CEI^c^ intervention (n=126)	Control (n=127)		
Age (y), mean (SD)	34.5 (9.9)	35.4 (10.6)	34.3 (10.6)	0.411 (2, 378)	.66
City, n (%)					
Mianyang	42 (32.8)	43 (34.1)	41 (32.3)	0.205 (4)	.96
Zibo	44 (34.4)	41 (32.5)	42 (33.1)		
Hohhot	42 (32.8)	42 (33.3)	44 (34.6)		
Current relationship, n (%)^d^					
Single	51 (39.8)	65 (51.6)	55 (43.3)	3.731 (2)	.16
Has a boyfriend	54 (42.2)	41 (32.5)	52 (40.9)	2.943 (2)	.23
Heterosexual marriage or has a girlfriend	17 (13.3)	12 (9.5)	18 (14.2)	1.424 (2)	.49
Divorced or widowed	13 (10.2)	11 (8.7)	8 (6.3)	1.260 (2)	.53
Education level, n (%)^e^					
Junior high school or below	17 (13.3)	11 (8.7)	9 (7.1)	6.156 (6)	.41
High school or associate degree	50 (39.1)	51 (40.5)	44 (34.6)		
Bachelor’s degree	38 (29.7)	43 (34.1)	48 (37.8)		
Master’s degree or above	15 (11.7)	16 (12.7)	11 (8.7)		
Employment status, n (%)					
Full-time	94 (73.4)	96 (76.2)	98 (77.2)	1.548 (6)	.96
Unemployed or retired	15 (11.7)	15 (11.9)	13 (10.3)		
Part-time	11 (8.6)	7 (5.6)	10 (7.9)		
Student	8 (6.3)	8 (6.3)	6 (4.7)		
Personal monthly income (CNY^f^), n (%)					
≤3000	28 (21.9)	35 (27.8)	23 (18.1)	4.141 (6)	.66
3001‐6000	55 (43.0)	52 (41.3)	61 (48.0)		
6001‐9000	31 (24.2)	28 (22.2)	28 (22.0)		
>9000	14 (10.9)	11 (8.7)	15 (11.8)		
Sexual orientation, n (%)					
Homosexual	101 (78.9)	101 (80.2)	97 (76.4)	2.347 (4)	.67
Bisexual or heterosexual	24 (18.8)	19 (15.1)	23 (18.1)		
Uncertain	3 (2.3)	6 (4.8)	7 (5.5)		

a An *F* test was conducted for Age. All other categories were analyzed with a chi-square test.

b HAPA: health action process approach.

c CEI: conditional economic incentives.

d Multiple selection allowed; percentages do not sum to 100%.

e Due to technical issues with the data collection system, some data on education level are missing.

f 1 CNY=US $0.145.

Furthermore, analyses of various personal and social characteristics revealed no significant group differences. This included educational attainment (*χ*²_6_=6.156, *P*=.41), current relationship status (*P*>.05), employment status (*χ*²_6_=1.548, *P*=.96), level of personal monthly income (*χ*²_6_=4.141, *P*=.66), and sexual orientation distribution (*χ*²_4_=2.347, *P*=.67). In terms of these characteristics, the descriptive profile shows that the majority of participants were either single or in a relationship, were primarily engaged in full-time employment, and reported a personal monthly income concentrated in the range of 3001 to 6000 CNY (US $436.02-$871.76). The predominant self-identified sexual orientation among participants was homosexual.

### Intervention Delivery and Follow-Up Status

Following the baseline assessment, the 3-month active intervention period commenced in January 2026. The intervention delivery, including tailored WeChat messaging, scheduled motivational interviews, and structured in-person group sessions for the HAPA+GCEI arm, is scheduled for completion in April 2026.

### Data Collection and Analysis Timeline

Data collection for the primary outcome and secondary outcomes is scheduled to conclude with the final follow-up assessment in August 2026, with full results expected to be published before December 2026. Database lock and statistical analysis are planned for December 2026.

## Discussion

This protocol outlines a study aimed at enhancing the uptake of HIV PrEP among Chinese MSM through a multifaceted approach, integrating the HAPA and conditional economic incentives. This research addresses the critical public health challenge of low PrEP use among high-risk populations, particularly MSM, who are disproportionately affected by HIV.

The HAPA model, a robust theoretical framework, provides a comprehensive strategy for understanding and promoting health behavior change. By focusing on both motivational and volitional phases, the HAPA model can effectively address the barriers to PrEP initiation and adherence [26]. This approach aligns with international research advocating for theory-driven, stage-matched interventions to bridge the intention-behavior gap, such as motivational interviewing for PrEP uptake among MSM in the United States [37]. However, many existing behavioral interventions in high-income settings do not adequately address the substantial financial barriers that are paramount in contexts like China, where PrEP is not covered by national health insurance [38]. This study directly responds to this gap by integrating HAPA with a pragmatic financial component.

Additionally, the incorporation of economic incentives represents an innovative strategy that leverages the power of financial rewards to encourage health-promoting behaviors. While economic incentives have shown promise in improving antiretroviral therapy adherence [39] and in pilot PrEP studies [33] elsewhere, our GCEI model innovates by applying a group-contingent structure within a Chinese MSM context. Economic incentives can reduce the financial burden associated with accessing PrEP, thereby overcoming one of the significant barriers to its use in the Chinese context [40]. By analyzing objective measures of PrEP initiation and adherence, this study will contribute valuable insights into the effectiveness of integrating behavioral interventions with economic incentives in promoting HIV prevention strategies, which is of global interest [41].

Several practical and operational considerations are crucial to ensuring the trial’s success. To maintain participant engagement and facilitate follow-up, providing clear and accessible information about the study and PrEP is essential. Regular reminders through various communication channels will be used to enhance retention rates. Furthermore, standard training for all staff involved in participant recruitment and data collection will be implemented to ensure high-quality and consistent data throughout the study.

However, this study does have potential limitations. First, the generalizability of the findings may be affected by the implementation through selected CBOs in specific urban Chinese settings, which may limit applicability across different cultural and socioeconomic contexts. The 3-month intervention period may not capture long-term behavioral patterns, suggesting that future studies should incorporate extended follow-up assessments. Additionally, the effectiveness of economic incentives could be influenced by external macroeconomic factors and individual attitudes toward financial rewards, though socioeconomic status will be included as a moderating variable in the analysis.

This study demonstrates several key strengths. An innovative intervention model has been designed by integrating eHealth technologies with established CBO partnerships. The WeChat-based platform is implemented in collaboration with 3 well-established MSM-friendly CBOs (each with 5‐10 y of documented HIV prevention experience), creating a potentially replicable implementation framework. The study design includes comprehensive pilot testing of behavioral change techniques prior to full implementation, with systematic application of HAPA theory across all intervention components. Both individual-level and group-level mechanisms have been carefully operationalized to allow for thorough evaluation. The hybrid intervention approach (combining digital and in-person components) addresses several implementation challenges. Existing social networks will be leveraged through CBO infrastructures, while privacy concerns will be mitigated through discreet eHealth communication channels. The protocol includes specific strategies for cost-effective scaling in resource-limited settings. These design features may provide valuable insights for bridging the intention-behavior gap in PrEP uptake among key populations.

In conclusion, this study outlines a comprehensive research protocol aimed at addressing the pressing challenge of low PrEP uptake among Chinese MSM. By integrating behavioral interventions based on HAPA with conditional economic incentives, we aim to identify effective strategies to enhance PrEP initiation and adherence in this vulnerable population. The outcomes have the potential to inform both public health practice in China and similar global settings by demonstrating how individual-level cognitive support and group-level financial strategies can be synergistically deployed within community-based and digital platforms to enhance HIV prevention.

## Supplementary material

10.2196/82123 Checklist 1 SPIRIT checklist.

## Abbreviations

CBO: community-based organization
GCEI: group-based conditional economic incentive
HAPA: health action process approach
LAI-PrEP: long-acting injectable-pre-exposure prophylaxis
MSM: men who have sex with men
PrEP: pre-exposure prophylaxis
SPIRIT: Standard Protocol Items: Recommendations for Interventional Trials
